# Tight Coupling of Na^+^/K^+^-ATPase with Glycolysis Demonstrated in Permeabilized Rat Cardiomyocytes

**DOI:** 10.1371/journal.pone.0099413

**Published:** 2014-06-16

**Authors:** Mervi Sepp, Niina Sokolova, Svetlana Jugai, Merle Mandel, Pearu Peterson, Marko Vendelin

**Affiliations:** Laboratory of Systems Biology, Institute of Cybernetics, Tallinn University of Technology, Tallinn, Estonia; Universidade Federal do Rio de Janeiro, Brazil

## Abstract

The effective integrated organization of processes in cardiac cells is achieved, in part, by the functional compartmentation of energy transfer processes. Earlier, using permeabilized cardiomyocytes, we demonstrated the existence of tight coupling between some of cardiomyocyte ATPases and glycolysis in rat. In this work, we studied contribution of two membrane ATPases and whether they are coupled to glycolysis - sarcoplasmic reticulum Ca^2+^ ATPase (SERCA) and plasmalemma Na^+^/K^+^-ATPase (NKA). While SERCA activity was minor in this preparation in the absence of calcium, major role of NKA was revealed accounting to ∼30% of the total ATPase activity which demonstrates that permeabilized cell preparation can be used to study this pump. To elucidate the contribution of NKA in the pool of ATPases, a series of kinetic measurements was performed in cells where NKA had been inhibited by 2 mM ouabain. In these cells, we recorded: ADP- and ATP-kinetics of respiration, competition for ADP between mitochondria and pyruvate kinase (PK), ADP-kinetics of endogenous PK, and ATP-kinetics of total ATPases. The experimental data was analyzed using a series of mathematical models with varying compartmentation levels. The results show that NKA is tightly coupled to glycolysis with undetectable flux of ATP between mitochondria and NKA. Such tight coupling of NKA to PK is in line with its increased importance in the pathological states of the heart when the substrate preference shifts to glucose.

## Introduction

In recent years, the compartmentalization of energetic processes has been a focus of bioenergetics research [1]–[3]. By studying SERCA activity dependence on ATP source [1], competitive inhibition of mitochondrial respiration by ATP-regenerating systems [4], [5], it has been demonstrated that there is a coupling between ATPases and mitochondrial respiration. This coupling could be explained by existence of intracellular diffusion restrictions [3], [6], [7]. However, these results could be influenced by inhomogeneity of skinned fiber preparation as well as possibly larger diameter of the fibers than cells. In addition to skinned fibers, it is possible that isolated cardiomyocytes – an alternative preparation to study intracellular bioenergetics – could be affected by clumping of cells and formation of micro-aggregates leading to increase of effective diffusion distances [8]. We recently tested this hypothesis by comparing cell population and single cell kinetics on isolated permeabilized rat cardiomyocytes [9]. The results show that diffusion restrictions do have an intracellular origin and are not an artifact of the cell preparation. Using an extension of raster image correlation spectroscopy for anisotropic media [10], Illaste *et al.* [11] measured diffusion coefficients for two fluorescent dyes in rat cardiomyocytes and, on the basis of the analysis of the measurements by mathematical model of intracellular diffusion, suggested the presence of an intracellular obstructive lattice that could cause restrictions in molecular movement. The nature of these barriers remains unknown and is a subject of active research [11].

Based on our earlier work on quantification of intracellular diffusion restrictions from kinetic measurements [3], [12], we developed an approach combining mathematical modeling and characteristic kinetic experiments to reveal aspects of intracellular compartmentation in rat cardiomyocytes [13]. Using that extended approach, we demonstrated that, in isolated permeabilized rat cardiomyocyte, diffusion is restricted by mitochondrial outer membrane and cytosolic diffusion obstacles grouping ATPases and mitochondria into a coupled system. Contribution of the both diffusion obstacles to the overall diffusion restriction obstructing the movement of ATP and ADP in the experiment was similar to each other. In addition, we demonstrated a strong coupling between endogenous PK and a fraction of ATPases in rat cardiomyocytes [13]. We have since applied the same protocol consisting of a set of characteristic kinetic experiments and several mathematical models on wild type and transgenic mice lacking the enzyme to produce creatine [14]. Interestingly, both of these mice models also suggest coupling between PK and ATPases. This shows that, although the activity of endogenous PK may vary, the coupling between some ATPases and glycolysis is present in the heart muscle cells of different mammalian species. Which ATPases are coupled to endogenous PK is not clear and requires further studies. By establishing the nature of the coupled ATPases, we would be able to predict which intracellular processes depend on glycolysis in predominantly oxidative cell, such as rat and mouse cardiomyocytes.

In order to specifically study the roles of different ATPases in cardiac energetics and establish which ATPases are linked to glycolysis, we turned to investigate membrane ATPases. The aim of the current work was to study whether sarcolemmal NKA or SERCA are active in permeabilized cardiomyocyte preparation and coupled to glycolysis. The relationship between sodium levels and aerobic glycolysis pointing to compartmentation of NKA and glycolytic energy production in heart has been in proposed in various studies [15], [16]. In the present work, we adopted the computational and experimental framework developed in [13] and used inhibitors of ATPases to quantify their activity, coupling to mitochondrial respiration, and glycolysis. Our results demonstrate that the NKA uses ATP generated exclusively by glycolysis with undetectable ATP flux between NKA and mitochondria.

## Results

The roles of SERCA and NKA in energetic compartmentalization in permeabilized relaxed rat cardiomyocytes was investigated. We found that, in our preparation, NKA is active allowing us to investigate it using an approach developed in [13]. The role of SERCA was found to be minor in our conditions.

### NKA activity

**Figure 1:**
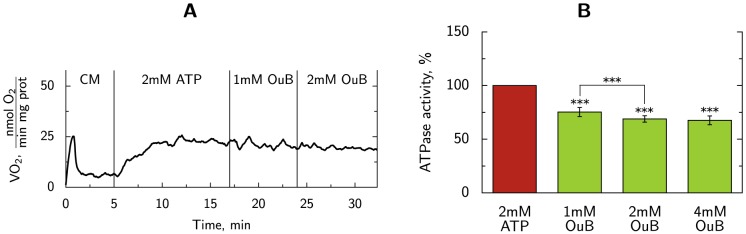
Contribution of NKA to overall ATPase activity. A: Representative example of respiration recording before and after NKA inhibition. In the plot, recorded respiration rate is shown after: cardiomyocyte (CM) addition giving the basal respiration rate, 2 mM ATP addition, and after addition of ouabain (OuB). Here, the vertical lines mark the time of ATP or inhibitor addition. B: Effect of NKA inhibition on total ATPase activity (*n* = 5) measured using PK+LDH system. Addition of 1 mM ouabain decreases ATPase activity by 25%, 2 mM ouabain and 4 mM ouabain reduced the total ATPase activity by 31% and 32% respectively. In these ATPase activity measurements, the ATP production by mitochondria is inhibited.

To study contribution of NKA, we used NKA inhibitor ouabain. When measuring the effect of ouabain on cellular respiration, we found that, in absence of ouabain, oxygen consumption rate stimulated by addition of 2 mM ATP was 22±6 nmol O_2_/min mg prot. Subsequent addition of 2 mM ouabain did not change the respiration rate significantly with the rate equal to 20±5.6 nmol O_2_/min mg prot (paired T-test, *p*>0.1, *n* = 7). In these experiments, exogenous ATP is hydrolyzed by cellular ATPases, with the produced ADP stimulating the cellular respiration. An example of an oxygraphic trace is shown in Fig. 1 A. To ensure that ouabain did not interfere with mitochondrial respiration, we performed kinetic tests with ATP and ADP which showed that respiration kinetics parameters in the presence and absence of ouabain are not statistically different (Table 1). Note that since there is no PCr or Cr in the solutions, absence of ouabain-induced effects on respiration could not be due to compensatory usage of PCr-related ATP pool.

**Table 1:** Respiration experiments results in NKA inhibited cells versus control cells.

Kinetic parameter	NKA inhibited	Control	p
Km(ADP), µM	844±203	692±175	0.3
Vmax(ADP), 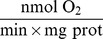	92±17	86±35	0.7
ACR(ADP)	8.3±1.4	8.1±2.06	0.5
	*n* = 7	*n* = 5	

To test whether ouabain reduces ATPase activity, we recorded how the addition of ouabain in different concentrations changes the total ATPase activity level achieved at 2 mM ATP. In these experiments, the total ATPase activity was measured via coupled PK+lactate dehydrogenase (LDH) system monitored in spectrophotometer through the change in NADH. To avoid competition between ATP-regenerating PK+LDH system and mitochondria, mitochondrial respiration was inhibited. We found that, in permeabilized cardiomyocytes, 2 mM ouabain reduced ATPase activity by 31±3% (Fig. 1B). This statistically significant (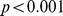) inhibition of total ATPase rate by ouabain clearly demonstrates that NKA is active in our preparation.

To test the dose-dependence on ouabain inhibition, we demonstrated that increasing the dose to 4 mM did not change the level significantly (Fig. 1B). This result confirms the reported maximal inhibition at ouabain concentration of 2 mM [17]. The same dose was used in the measuring solution when the following kinetic experiments for the mathematical modeling were performed.

To study the interaction between NKA and glycolysis, we tested the effect of ouabain on total ATPase activity in the absence of glucose. In this experiment, glycolysis was inactive due to the absence of glucose in the cells' storage solution (wash solution). We found that, in the absence of glucose, there was no ouabain effect on ATPase activity measured via coupled PK+LDH system. ATPase activity in glucose free cells stimulated by 2 mM ATP remained the same after 2 mM ouabain addition: 148±43 nmol/(min mg prot) in the absence of ouabain and 143±47 nmol/(min mg prot) after 2 mM ouabain addition (paired T-test, 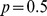, *n* = 6). Thus, to activate NKA, ATP has to be provided by a chain of endogenous glycolysis reactions (which are inactive in the absence of glucose) and it is not sufficient to provide exogenous PEP, as in PK+LDH system used in these experiments. From the absence of ouabain inhibition effect on overall ATPase activity, we conclude that NKA is tightly coupled to the part of the glycolytic system that is localized in the vicinity of NKA, i.e. in the subsarcolemmal space.

### Analysis of compartmentation in NKA inhibited cells

**Figure 2:**
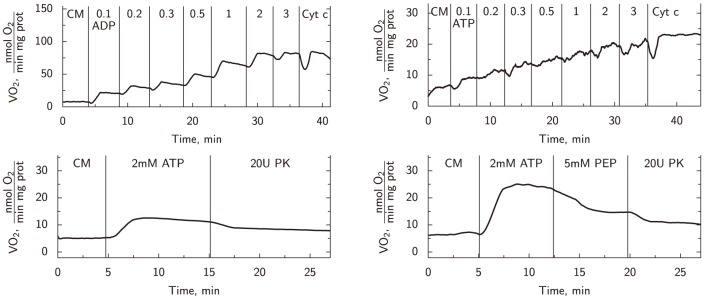
Representative examples of respiration experiments on NKA inhibited cells. CM indicates the basal respiration after a cardiomyocyte (CM) suspension was introduced, vertical lines mark the time of metabolite or inhibitor addition. The top row shows an example of the ADP titration (left) and ATP titration (right), vertical lines mark the time of introduction of ADP or ATP at indicated millimolar concentrations into the solution. The bottom row demonstrates inhibition of respiration initiated by 2 mM ATP addition of 5 mM PEP and 20 U/ml PK into the system. Two cases were considered. On the bottom left, PEP was present in solution before addition of 2 mM ATP. On the bottom right, PEP and PK were added consecutively.

**Figure 3:**
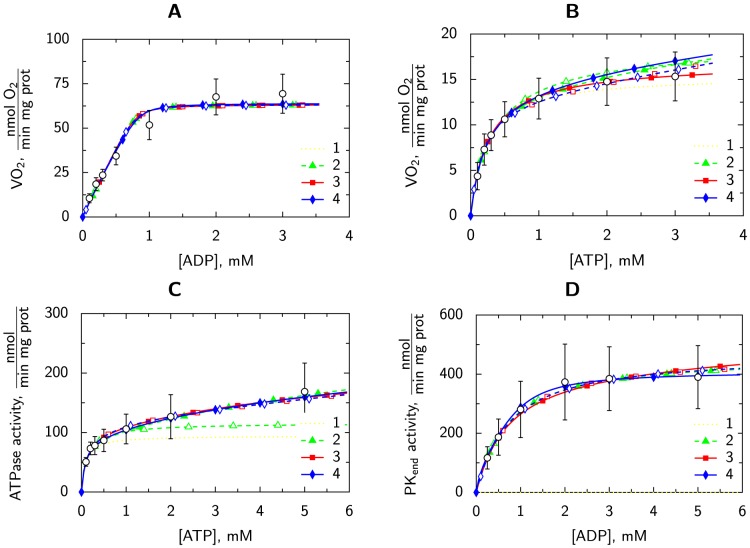
Experimental data recorded on NKA inhibited cells (open circles, mean ± STD) are compared to the calculated model solutions. Obtained model solutions are shown by solid lines with filled symbols for models 1–4 and dashed lines with open symbols for the simplified versions of the models (2s, 3s and 4s). The experimental data from respiration experiments: oxygen consumption rate recorded during titration with ADP (A) or titration with ATP (B); data from spectrophotometric experiments: total ATPase activity (C), and endogenous PK activity (D). In addition, since there is no endogenous PK activity in model 1, the rate of PKend calculated by model 1 is zero in D. Note that all of the models that take into account endogenous PK (models 2–4) produce similar fits with no model fit being conclusively superior to the others.

To determine the level of coupling of NKA with ATP-generating systems, we completed a full set of characteristic kinetic experiments consisting of four oxygraphic and two spectrophotometric measurements on NKA inhibited cells, as described in Methods. Representative graphs of the respiration experiments on NKA inhibited cells are given in Fig. 2. The kinetic measurements, summarized in Fig. 3 and Table 2, were complemented with the mathematical analysis using seven different mathematical models with varying cyto-architectural and functional complexity.

**Table 2:** Respiration rate stimulated by endogenous ATPases and inhibited by PK recorded on NKA inhibited cells.

	Experimental	1	2	2s	3	3s	4	4s
VO_2_ (PEP), nmol/min mg prot	7.97±2.09	**13.44**	7.2	7.0	6.97	7.37	6.86	7.36
VO_2_ (PK+PEP), nmol/min mg prot	2.76±1.15	2.87	3.74	3.67	3.61	3.66	3.54	3.65

Respiration rate was stimulated by endogenous ATPases and inhibited by endogenous PK (VO_2_ (PEP)) only or together with exogenously added 20 U/ml PK (VO_2_ (PEP+PK)). The experimental data are compared with the simulation results obtained by the full models 1, 2, 3, 4 and their corresponding simplified versions (2s, 3s, and 4s). Endogenous ATPases were stimulated by 2 mM ATP and PK was activated by 5 mM PEP added to solution. Shown in bold the optimization result that differs from the experimental value more than two standard deviations.

**Figure 4:**
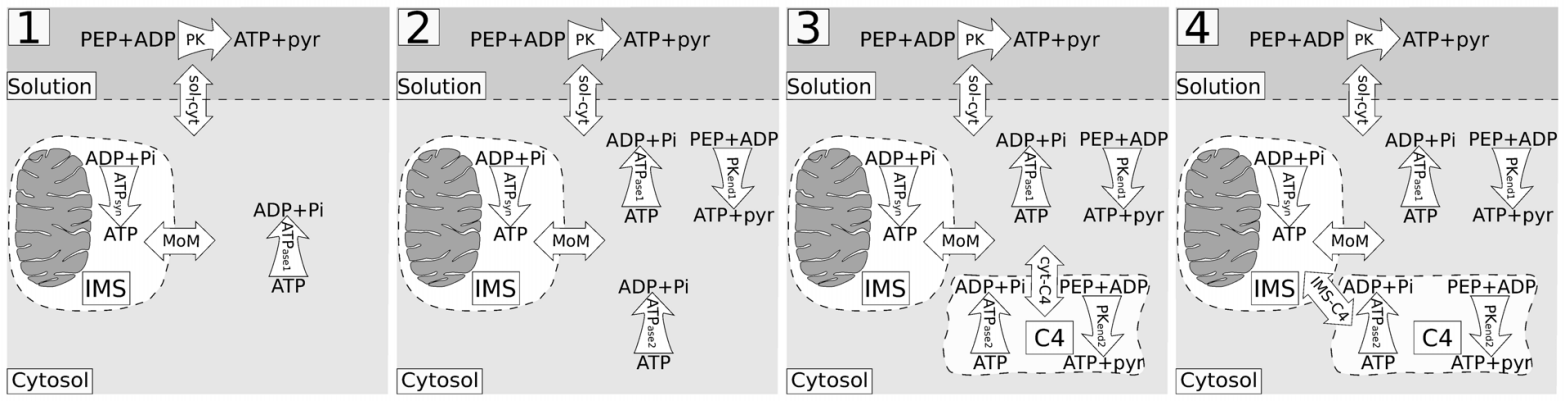
Models of different spatial organization and reactions in permeabilized cardiomyocytes. The compartments considered in different model versions: Solution, Cytosol, IMS, and an additional fourth compartment C4. The simplified description of ATP synthase taking place in IMS is justified by the experimental conditions with high concentrations of Pi, oxygen and substrates. The processes accounted for, noted with curved arrows are: ATP synthesis (ATPsyn), ATP consumption (ATPase_1,2_), exogenous (PK) and endogenous (PK_end1,2_) pyruvate kinase reaction; exchange of metabolites between compartments is marked with doubled-headed arrows.

Here, four model versions having different intracellular compartmentation levels with specific reactions defined in each sub-compartment were considered (Fig. 4). The model solutions fitted to the experimental kinetic data are show in Fig. 3 and to PK-induced inhibition in Table 2. The optimal parameter values together with their confidence intervals are given in Table 3. Almost equally good fits were obtained with all considered models, with the exception of Model 1. Model 1 is not able to reproduce the endogenous PK activity data due to the absence of this reaction in the model.

**Table 3:** Model parameters found by fitting the experimental data recorded on NKA inhibited cells.

Model	1	2s	2	3s	3	4s	4
**V** 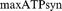 , 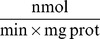	380	380	380	380	380	380	384
	147–721	293–479	287–487	290–484	276–501	290–484	281–511
**C** 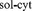 , 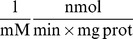	3126	2727	3151	2011	2157	1992	1412
	527–999998	1206–10307	1242–17624	863–7542	851–10083	856–7463	544–6999
**C** 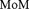 , 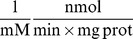	558	574	559	610	606	611	612
	240–1126	446–724	430–711	461–791	442–810	461–793	422–865
**C** 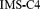 , 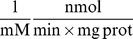						7.4	527
						0.014–17	233–1404
**C** 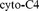 , 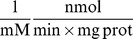				7.59	0.872		
				0.013–17	0.393–54		
**V** 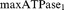 , 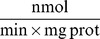	95	116	367	81	102	80	372
	56–138	99–133	48–753	68–95	84–121	67–95	98–728
**K** 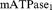 , **mM**	0.053	0.087	10	0.058	0.068	0.057	9.99
	0.05–0.248	0.056–0.133	4.47–10	0.05–0.106	0.05–0.12	0.05–0.104	4.76–10
**K** 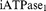 , **mM**	0.05	0.05	0.05	0.085	0.05	0.079	0.05
	0.05–10	0.05–10	0.05–10	0.05–10	0.05–0.355	0.05–10	0.05–0.224
**V** 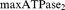 , 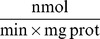			87	4410	183	4500	88
			71–103	13–4418	0.0–486	14–4500	70–107
**K** 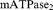 , **mM**			0.051		9.46		0.051
			0.05–0.094		2.51–10		0.05–0.097
**K** 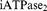 , **mM**			0.05		0.051		0.051
			0.05–10		0.05–10		0.05–10
**V** 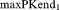 , 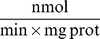		460	461	421	395	420	0.329
		322–605	313–615	276–575	239–564	274–574	0.149–102
**K** 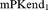 , **mM**		0.614	0.625	0.64	0.533	0.641	0.164
		0.315–1.25	0.305–1.36	0.343–1.28	0.214–1.41	0.345–1.28	0.05–0.513
**V** 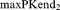 , 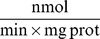				44	6925	46	410
				2.35–87	0.0–6953	3.71–88	250–605

The calculated parameter values are shown above the confidence interval for each model parameter.

To quantify the difference in fits for all model versions, we performed a statistical analysis of the numerical solutions using various information criteria and an F-test of nested models. The results of the statistical analysis are given in Table 4. According to the analysis, the likelihood that model 1 is correct and all the more complicated models produced better fits by chance is very small (nested F-test, *p*<0.0001). By comparing full models to their corresponding simplified models, we can see that the usage of two sets of apparent affinity constants is not justified. AIC criteria places models 3s and 4s above 2s (the best fit has the smallest criterion value). However, AICc and BIC criteria that penalize the larger number of parameters more than AIC, indicate model 2s as the best with smallest AICc and BIC scores. The criteria AICc and BIC for 3s and 4s were relatively small and not too far from the value obtained for 2s. When comparing the models using the nested F-test, *p* -values indicate that the better fits obtained by models 3s and 4s when compared with the fit obtained by the model 2s could be due to chance (*p* values are 0.065 and 0.067, depending on the models compared). Because these values are close to the cutoff value of *p* = 0.05, we cannot fully exclude models 3s and 4s from the following analysis and conclude that there are no ATPases tightly coupled to endogenous PK after inhibition of NKA (the main difference between models 2s and 3s together with 4s). However, the reduction of significance in F-test when comparing control [13] to NKA-inhibited (Table 4) case suggests that the contribution of ATPases tightly coupled to endogenous PK is smaller in NKA-inhibited case than in control.

**Table 4:** Statistical analysis of model fits for NKA inhibited cells.

					F test					
Model	AIC	AICc	BIC	Residual	2s	2	3s	3	4s	4
**1**	41.28	45.1	99.81	79.60	<0.0001	<0.0001	<0.0001	<0.0001	<0.0001	<0.0001
**2s**	−28.26	−21.06	33.24	6.30		0.114	0.065	0.237	0.067	0.183
**2**	−31.59	−16.07	41.61	4.57				0.58		0.409
**3s**	−33.6	−18.07	41.3	4.26				1.0		
**3**	−29.57	−5.3	48.04	4.27						
**4s**	−33.52	−17.99	41.32	4.28						0.695
**4**	−30.83	−6.57	47.86	4.09						

Models in rows are compared with more complicated model version in columns and F-test *p* value is shown.

**Figure 5:**
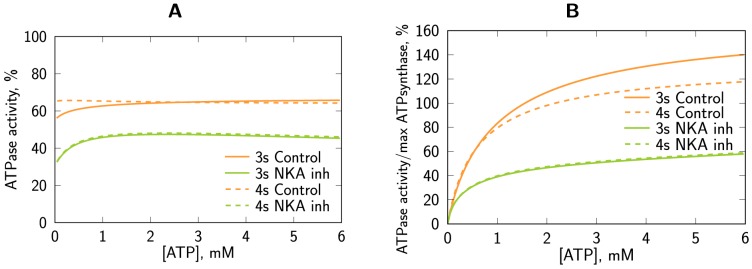
ATPase activity in compartment C4 relative to total ATPase activity at given ATP concentration (A), or related to maximal ATP synthase rate (B). Regardless of the used model, NKA inhibition reduces the relative activity in compartment C4. Here, the ATPase activities are calculated with inhibited respiration and regeneration of ATP by exogenous and endogenous PK, as in the experiments performed in spectrophotometer in this work.

To analyze compartmentation of NKA-inhibited cells further, we compared the control case analyzed earlier [13] with the results of our analysis in this work. As we showed in [13], in the control case, with all ATPases working, the results clearly favored models 3s and 4s with an additional compartment C4. Direct comparison of kinetic parameters for ATPases and PK in compartment C4 is not that informative because, as discussed in [13], the description of coupling between ATPases and PK in C4 is phenomenological. However, regardless of this phenomenological description, we can compare ATPase rates calculated by the models to compare the kinetics of ATPases at different conditions. For this comparison, we used the simplest models that incorporated coupling between ATPases and PK in C4 (models 3s and 4s). In the control case, regardless of the model used, about 60% of total ATPase activity occurs in C4 when performing the simulations that correspond to the measurement of ATPase activity in spectrophotometer with inhibited mitochondrial respiration. In contrast, for NKA-inhibited cells, C4 accounts for only about 40% of the total ATPase activity (Fig. 5A). As demonstrated in Fig. 5B , in general, the kinetics of ATPases in C4 was similar in control and NKA-inhibited cells with the exception of the maximal ATPase rate. At all ATP concentrations used, inhibition of NKA reduced the C4-bound ATPase rate by approximately half of that of the control cells. Thus, according to our results, inhibition of NKA reduces the ATPase activity mainly in the compartment C4 representing ATPases tightly coupled to endogenous glycolysis.

### SERCA activity

**Figure 6:**
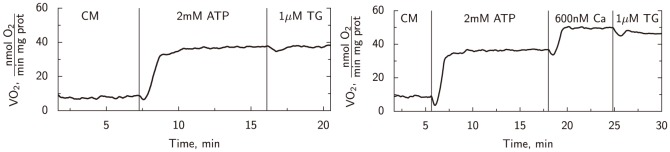
Representative example of experimental recording showing the role of SERCA in mitochondrial respiration. In both plots, vertical lines mark the introduction of a new agent indicated right of the line. First, the cardiomyocyte suspension (CM) is introduced, after a stable basal O_2_ consumption is achieved, respiration is activated by 2 mM ATP via endogenous ATPases. In the first experiment (left), the respiration rate is unchanged after inhibition of SERCA by addition of 1 µM TG. In the second experiment (right), increasing the free Ca^2+^ to 600 nM gives approximately 50% rise in oxygen consumption. Subsequent addition of 1 µM TG reduces the achieved respiration rate less than 10%.

The role of SERCA was investigated by checking how the energy production and consumption processes are affected by SERCA inhibition. We found that, in Ca^2+^ free conditions (the standard setup in our studies), introduction of SERCA inhibitor TG did not alter the respiration level (see representative oxygraphic trace in Fig. 6, left). In addition, no reduction in total ATPase activity levels was recorded after SERCA inhibition. These experiments demonstrated that SERCA is inactive in our conditions and its role in intracellular compartmentation cannot be assessed in this preparation. Further measurements of SERCA's role were performed in elevated free Ca^2+^ situations in both respiration and spectrophotometric measurements confirming the minor role of this ATPase in our cell preparation (File S1).

## Discussion

In this work, the properties of SERCA and NKA energy consumers were studied via mitochondrial ATP production and cytosolic ATP consumption measurements at different metabolite levels. We found that, in our experimental conditions with permeabilized sarcolemma and nominal Ca^2+^ -free medium, NKA plays a substantial role in overall ATPase activity. Our main result is a demonstration of tight coupling between NKA and endogenous glycolysis. Namely, all ATP fueling NKA was shown to come from glycolysis with the other sources — mitochondria and exogenous ATP — not used by NKA. In addition, our analysis of NKA participation in bioenergetics of the heart muscle cell have demonstrated that isolated permeabilized cardiomyocytes are a promising preparation for studies of membrane ATPases.

### NKA measurements and results

To interpret our results, we have to clearly understand what do we measure. One of the key experiments demonstrating tight coupling between NKA and endogenous glycolysis is the determination of total ATPase activity in the cell. In order to establish total ATPase activity, we inhibit mitochondrial respiration and introduce ATP-regenerating system – PEP for the PK reaction, together with PK, LDH and NADH. Using spectrophotometer we measure the changes of NADH concentration in solution. These changes are attributed to LDH reaction which uses pyruvate and NADH as substrates. Since there is no pyruvate in solution, NADH usage by LDH is directly linked to pyruvate production by PK as a result of ADP to ATP conversion by PK. Taking into account the high activity of added PK and LDH when compared to the activities of ATPases in the cells in solution and high degree of irreversibility of reactions, NADH concentration changes follow ATPase activity in solution. Our measurements demonstrate that when glucose has been washed away from the cells by removing it from the used solutions, there has been no effect of ouabain on total ATPase activity when measured in spectrophotometer. In other words, pyruvate production does not depend on NKA if there is no glucose. Thus, NKA uses ATP provided by a part of glycolysis system that is tightly coupled and does not accept PEP from surrounding solution as a substrate. Otherwise, we would have observed ouabain-induced inhibition of total ATPase activity in the absence of glucose.

**Figure 7:**
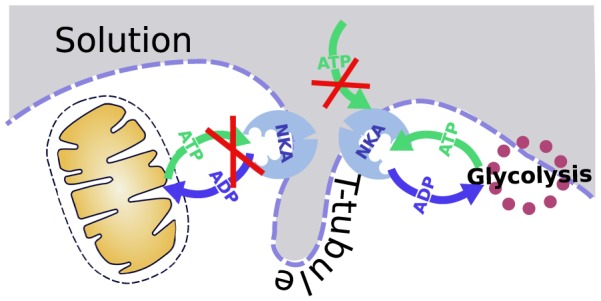
Scheme of NKA compartmentalization proposed on the basis of our analysis. ATP from glycolytic origin is preferentially used by NKA over mitochondrially produced ATP and exogenously added ATP.

Our results on compartmentation of NKA energy supply in the isolated permeabilized heart cells are summarized in Fig. 7. Inhibiting NKA has no effect on respiration stimulated by exogenous ATP or ADP. Thus neither ATP from the solution nor ATP generated by the mitochondria is used by the NKA. Note that there is no PCr in the solution to potentially offer ATP to NKA and complicate the interpretation of our recordings. On the other hand, inhibition of NKA significantly reduced total ATPase activity. As discussed above, on the basis of total ATPase activity measurements, we demonstrated that NKA used only ATP supplied by endogenous glycolysis (Fig. 7).

In our earlier work, we demonstrated that there are ATPases which are tightly coupled to endogenous glycolysis [13]. Those ATPases were simulated in mathematical models by introducing compartment C4 (Fig. 4, models 3 and 4) and making them active in this compartment. As established in the present work, one of these ATPases is NKA. Our analysis also revealed relative activity of NKA by comparing the model solutions for control and NKA-inhibited case (Fig. 5). NKA inhibition reduced ATPase activity only under conditions where ATP was provided by endogenous glycolysis, i.e. the activity of ATPases confined to compartment C4 was reduced (Fig. 5). Various statistical criteria and an F-test of fits of nested model pairs were used to interpret the modeling results. As a result of the ouabain induced decrease in ATPase activity in C4 compartment, the statistical evidence confirming the existence of the compartment C4 was significantly smaller when compared to the control case reported in [13]. Such reduction of statistical evidence of ATPase–endogenous glycolysis coupling existence clearly demonstrates that NKA is one of the ATPases coupled to endogenous glycolysis and its inhibition leads to decrease of signal to noise ratio in the experiments suggesting the existence of ATPase–glycolysis coupling. Taking the measurements and analysis together, we conclude that NKA is active in our preparation and is tightly coupled to glycolysis.

Despite the demonstrated tight connection between glycolysis and NKA, the precise glycolytic reaction producing the ATP for NKA cannot be determined on the basis of the performed experiments and would require extensive further studies. Theoretically, ATP used by NKA can be provided by two glycolytic reactions: one catalyzed by phosphoglycerate kinase and the other by PK. Studies on erythrocytes found that NKA is coupled to phosphoglycerate kinase [18], [19]. Whether or not this holds for cardiac cells remains to be investigated. There are differences in PK isoforms with contradistinctions in allosteric properties towards PEP [20], [21] in heart and red blood cells that may play a role in coupling between NKA and glycolysis enzymes.

### Comparison with earlier studies

Dominant role of glycolysis in providing energy for the membrane processes has been known for decades. Detailed studies of the glycolytic pathway which specifically demonstrated connection to NKA were performed in red blood cells [19], [22]. The preference of glycolysis-supplied ATP for sarcolemmal function has been shown in isolated perfused heart [15], [23], [24], smooth muscle [25], [26] and intact skeletal muscle [27], [28]. In intact muscle preparations and perfused heart, studies of NKA-glycolysis coupling were performed by modulation of energetic pathways or NKA activity. Energetic pathways have been modulated by inhibiting glycolysis and/or oxidative phosphorylation [15], [24]. To alter NKA activity several approaches were used: inhibition by ouabain or by incubation in Na^+^-free medium [28]–[30]; stimulation by increase in Na^+^ concentration induced by Na^+^ ionophores [30], [31]. These interventions of energetic pathways or NKA activity can lead to problems in drawing conclusions since there could be several side-effects. Namely, the inhibitors are often not specific, alter intracellular pH that in turn affects glycolysis, cause buildup of potentially toxic glycolytic intermediates and change membrane permeability to Na^+^, compromising the NKA investigation. Thus, despite the fact that intact preparation in many respects corresponds closely to *in vivo* situation, the manipulation and control of experimental conditions in intact cell is difficult. Often considerable experimental effort is needed to prove that the observed effects are indeed due to the direct intervention and are not caused by undesired secondary effects. We have found that, in isolated permeabilized cardiomyocyte with the intracellular medium easily controllable, the NKA pump preserves its activity on the cell membrane even in conditions where the integrity of plasmalemma has been compromised by permeabilization. This demonstrates that this preparation could be a promising tool for investigation of NKA.

In this work, where we combined experimental data with mathematical modeling, we demonstrate that NKA is solely fuelled by glycolysis. This exclusivity of glycolytic energy production is a point where previous reports left some ambiguity. For example, studies of inside out vesicles from human red cell membranes reported stimulation of NKA by the exogenous ATP [22]. In tissues where both glycolytic and oxidative pathways are available, the concentration of Na^+^ in the medium and the used substrates are important factors in evaluation of the pathway's coupling to NKA. At low Na^+^, aerobic glycolytic metabolism supports the NKA with additional support of the oxidative metabolism needed only at higher NKA working rates [26]. Our finding that NKA uses endogenous glycolysis as a source for ATP is in agreement with the reported inhibition of NKA when glucose is unavailable [27] and NKA preference for glycolysis-supplied ATP suggested on the basis of glycolysis and mitochondrial respiration inhibition [24].

### Membrane ATPases are exposed in permeabilized cells and fibers

The high activity of plasmamembrane-localized NKA observed in this study has important consequences regarding the interpretation of kinetic measurements performed on permeabilized cells and fibers. Kinetic measurements of oxidative phosphorylation on permeabilized fibers and cells are frequently used to study intracellular compartmentation and the permeability of the mitochondrial outer membrane in different cells from animals and human preparations [32]–[38]. Our results show that the membrane permeabilization can lead to a high flux of sodium and potassium induced by NKA and, possibly, maximal activation of NKA under certain conditions. This high activity of NKA, and possibly other plasmamembrane ATPases, should be taken into account when analyzing measurements performed on permeabilized preparations and when drawing conclusions regarding intracellular compartmentation in health and disease.

### Physiological role

Sarcolemmal NKA pumps sodium out and potassium into the cell. NKA contributes to the sodium balance and cardiac contractility via the Na^+^/Ca^2+^ exchanger (NCX) that uses the sodium gradient to move calcium. The activity of NKA is rather high and, in sarcolemmal vesicles obtained from Ca^2+^ -free perfusion of rat heart, was about 30–50% of the total activity of all ATPases [39]. We observed a 31% decrease in total ATPase activity when we inhibited NKA with 2 mM ouabain. This suggests that the functioning of NKA in permeabilized cardiomyocytes is closely matched to its operation in the whole organ level in calcium-free conditions.

The NKA contributes 5–20% of the basal metabolism (usage that is not electrical activation or mechanical contraction) in a contracting heart [40], [41]. NKA participates in the calcium handling via NCX, with calcium, in addition to regulation of many processes in the cell, modulating the glycolysis [42]. In resting state, phosphofructokinase and PK are under feedback control by PCr and ATP [43]. Analyzing the energetics with no PCr and calcium, as done in this study, can artificially amplify the contribution of the NKA-glycolysis coupling. However, it is reasonable to assume that the conclusions of this coupling apply to the intact beating cells as well. The only question is the extent of how this coupling contributes to the vast dynamic system of reactions in intact beating cell *in vivo*. In the intact beating cells, the interplay of reactions is more complex as both Ca^2+^ and PCr that affect contraction, also affect activity of different glycolytic enzymes [44], [45]. As a continuation of our study, the various activators and inhibitors of the PK system [43]–[47] as well as PEP concentration dependency have to be considered to understand the interaction between NKA and glycolysis *in vivo*.

Tight coupling between NKA and glycolysis is in accordance with the results reported by others. It supports the view of multiple functional cytosolic pyruvate pools [48] one of which is connected to NKA. The coupling between NKA and glycolysis demonstrated by us confirms that energy produced via glycolysis is preferentially used to support sarcolemmal function [15]. Tight coupling between NKA and glycolysis demonstrated in our preparation has important physiological implications. Assuming that the same coupling persists in pathological cases, such as late stages of heart failure, this coupling is in agreement with the shift of substrate utilization towards glycolysis [49] and, in heart failure models, shift of Ca^2+^ handling from SERCA towards NCX [50] that is kept in balance by NKA. As a model of heart failure, the changes in calcium handling in conditional cardiomyocyte-specific SERCA2 knockout have been recently analyzed by data-driven mathematical models of calcium dynamics [51]. According to this analysis, the development of end-stage heart failure correlated with the increase of NCX contribution to calcium removal and, as a result, increased ATP consumption by membrane transport. In addition, while ATP consumption per cardiac cycle was similar for SERCA and NKA in control, the development of end-stage heart failure changed the distribution of ATP consumption towards dominant role of NKA in using ATP for Ca^2+^ handling [51]. Assuming that the same tight coupling between PK and NKA persists in this animal model, such shift towards dominant role of NKA could lead to high load on glycolysis in cardiomyocytes and, ultimately, to energetic imbalance and development of end-stage heart failure, as suggested in [51].

### SERCA is inactive in our preparation

In addition to NKA, we briefly analyzed the activity of SERCA in our preparation. In agreement with low calcium concentration used in our solution, SERCA activity was undetectable. Thus, we could not analyze the interaction between SERCA and ATP-regenerating systems in the heart using our approach. However, when SERCA loading was analyzed by Kaasik et al [1], it was found that ATP regeneration by mitochondrial respiration was very efficient when compared to exogenous ATP supply in permeabilized fibers. These results suggested that SERCA and mitochondria are surrounded by intracellular diffusion restrictions leading to formation of a functional unit that has ATPases and ATP-regenerating system - mitochondria.

### Conclusions

In summary, high degree of coupling between NKA and glycolysis demonstrated in this work and coupling between SERCA and mitochondrial oxidative phosphorylation shown earlier by others [1] suggest that substrate utilization and calcium handling in the heart is tuned to ensure, as much as possible, sufficient energy supply in health and disease to calcium handling currents by forming coupled SERCA-mitochondrial oxidative phosphorylation and NKA-glycolysis pairs.

## Materials and Methods

### Experimental procedures

Adult outbred Wistar rats of both sexes weighing 300–500 g were used in the experiments. Animal procedures were approved by Estonian National Committee for Ethics in Animal Experimentation (Estonian Ministry of Agriculture).

Before harvesting the heart, the animals were anesthetized with an i.p injection of 0.5 mg/ml dexmedetomidine (Dexdomitor; Orion, Espoo, Finland) and 125 mg/ml ketamine (Bioketan, Vetoquinol Biowet, Gorzów Wielkopolski, Poland). The calcium tolerant cardiomyocytes were isolated using Langendorff perfusion following the procedure described previously in [13] with slightly attuned composition of solutions as listed in [9] and below to give a concise overview.

#### Solutions

Wash solution in (mM): 117 NaCl (Sigma-Aldrich, 71379), 5.7 KCl (Sigma-Aldrich, P5405), 4.4 NaHCO_3_ (Sigma-Aldrich, S6014), 1.7 MgCl_2_ (Sigma-Aldrich, 63068), 1.5 KH_2_PO_4_ (Sigma-Aldrich, P0662), 21 HEPES (Sigma-Aldrich, H3375), 20 Taurine (Sigma-Aldrich, 86329), 11.7 Glucose (Sigma-Aldrich, 158968). pH was adjusted to 7.4 with NaOH at 25°.

The composition of the digestion solution was the same as the wash solution with the addition of 3 mg/ml BSA (Roche, 10 775 835 001), 10 µM Leupeptin (Roche, 11 034 626 001) and 2 µM soybean trypsin inhibitor (Fluka, 93619) and collagenase P (Roche). Composition of sedimentation solution was the same as digestion solution without the collagenase. Calcium tolerance was achieved by introducing CaCl_2_ into the sedimentation solution in the final concentration of calcium up to 1 mM.

Measurement (Mitomed) solution (mM): 3 KH_2_PO_4_, 3 MgCl_2_, 110 Sucrose, 60 K-lactobionate, 20 Taurine (Sigma-Aldrich, 86329), 20 HEPES, 0.5 EGTA, 0.5 DTT, 2 Malate, 5 Glutamate and 5 mg/ml BSA (Roche, 10 775 835 001). pH was adjusted to 7.1 with KOH at 25°.

Note that while there is no Na^+^ in measurement solution, Na^+^ is transferred into the medium by moving the cells kept in Na^+^-rich extracellular solution used during isolation, into the measurement solution. Due to the large concentration of Na^+^ in extracellular solution, we estimated that in our experimental conditions during the measurements, the concentration of Na^+^ was in a range of 1.2–3.6 mM.

#### Activity measurements

The effect of two membrane ATPases - SERCA and NKA, was assessed by investigating how inhibition of one selected ATPase changes mitochondrial respiration and total cellular ATPase activity measured spectrophotometrically on saponin (25 µg/ml, Sigma 47036) permeabilized cardiomyocyte suspension at 25°C. NKA was inhibited with 2 mM ouabain (O3125 Sigma) [17], [39]. SERCA was inhibited with thapsigargin (TG, Asc-286 Ascent) over the concentration range of 0.1–1.1 µM [52], [53]. The effect of thapsigargin was also tested in elevated physiological range of free Ca^2+^ concentrations of 100, 200 and 600 nM [53], [54]. Free calcium concentrations were calculated using MAXC [http://www.stanford.edu/~cpatton/maxc.html]. Spectrophotometric experiments were performed with a Thermo Evolution 600 (Thermo Scientific, Waltham, MA). The cytosolic total ATPase activity and endogenous PK activity was assessed by monitoring changes in the concentration of NADH through a coupled lactate dehydrogenase-PK assay in the absence of mitochondrial respiration. Oligomycin (10 µM, Sigma-Aldrich 75351) and sodium cyanide (5 mM, Sigma-Aldrich 205222) was used to inhibit oxydative phosphorylation. Respiration rate and spectrophotometrically measured activity rates were normalized to protein concentration determined by Bradford assay (Fermentas 1271) or by a Nanodrop 2000 (Thermo Fisher Scientific Inc.) measurement using BSA as a standard in both. The interchangeability of these two methods was verified by a paired T-test.

#### Experiments for mathematical modeling

The data used as input for the mathematical models came from six different experiments performed using the protocols described in [13]. In short: the dependence of oxygen consumption rates on the incremental increase of ADP and ATP concentrations was measured. The effect of consecutive addition of 5 mM PEP and 20 IU PK on ATP-initiated respiration was also recorded. In this experimental series respiratory experiments were performed with a Strathkelvin 6 chambered respirometer (Strathkelvin Instruments RC650 Respirometer, North Lanarkshire, Scotland). Real time oxygen consumption rates were calculated using the IOCBioStrathKelvin software available at http://iocbio.googlecode.com. In addition, extramitochondrial kinetics of ATPases and endogenous PK were also recorded spectrophotometrically. The lactate dehydrogenase-PK coupled assay was used on NKA inhibited cells to monitor the changes in total ATPase activity at stepwise increases in ATP, and endogenous PK activity response to incremental increase in ADP concentration.

Mitochondrial respiration was inhibited with oligomycin and cyanide for spectrophotometric recordings.

#### Statistics

The raw data was analyzed using custom-made software. All results are shown as mean ± std. dev. The apparent Km values were estimated by fitting the data with Michaelis Menten equation. Statistical comparisons were made using F- and T-tests using the following notation for statistical significance: */#: 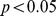, **/##: 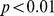, ***/###: 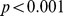.

#### Mathematical model

The detailed descriptions of the mathematical models used in the analysis are given in [13]. In brief, the models describe ATP production and utilization processes and implement four different intracellular compartmentation schemes, depicted in Fig. 4. The compartments considered in all of the model versions were: extracellular solution, cytosol, and the intermembrane space (IMS). In models 3 and 4, an additional compartment C4 was added into the cytosol. Mitochondrial ATP production was described using a simple phenomenological Michaelis-Menten-type equation depending on ATP and ADP concentrations in the IMS only, the intricacies of electron transfer chain on the inner mitochondrial membrane were omitted in this implementation. A simplified model of ATP synthase taking place in IMS is justified by the experimental conditions with high concentrations of Pi, oxygen and substrates. The processes considered in the models were ATP synthesis by mitochondria, ATP hydrolysis by ATPases, endogenous pyruvate kinase reactions that occur in the cytosol and in compartment C4, exogenous pyruvate kinase reaction in solution, and the diffusion of metabolites between compartments. For models 2, 3 and 4 we created additional modified versions of these models keeping the compartmentation structure but applying constraints on the optimized kinetic parameters. In models 2, 3, and 4, the ATPase reactions or cytosolic PK reactions are divided into two parts with two sets of apparent affinity parameters. In the simplified versions of these models (termed 2s, 3s and 4s), the apparent affinity parameter values for the same type reactions were assumed to be equal.

#### Statistical comparison of modeling results

Introduction of additional parameters to the models, in general, leads to a better fit of the model solution to experimental data. However, it has to be evaluated whether or not the increased number of parameters is justified or if the better fit is merely a result of increased degrees of freedom without adding quantitative insight to the interpretation. The comparison between models wa achieved by evaluating the goodness of fit for every model version using the Akaike Information Criteria (AIC), corrected AIC (AICc) and the Bayesian Information Criteria (BIC). These criteria enable one to list the models based on the residual reached. The smaller the criteria value, the better the model.

In addition to multiple information criteria, the models were compared by F-test of nested models (models 1, 2, 3 and 1, 2, 4). Note that by reducing processes, model 1 can be derived from models 2, 3 and 4. However, models 3 and 4 are not nested. The *p*-value of the F-test of two nested models gives a probability that the simpler model was sufficient and a larger residual was obtained by chance.

To assess the sensitivity of each model, confidence intervals for all model parameters were estimated. For that, every calculated value in a set of optimal parameters was tested in the following way: one parameter value was allowed to change whereas the other found parameter values were kept at their optimal values. This change in the selected parameter resulted in a worse fit. As soon as the *p* -value for the extra sum-of-squares F-test comparing new fit and the optimal fit became 0.05, the procedure was stopped and the parameter value was recorded at the lower or higher end of the confidence interval depending if the selected parameter value was allowed to decrease or increase.

## Supporting Information

File S1 **Supporting material.**
(PDF)
