# Supplementary material for: Tight Coupling of Na+/K+-ATPase with Glycolysis Demonstrated in Permeabilized Rat Cardiomyocytes
Source: PLoS One. 2014 Jun 16;9(6):e99413. doi: 10.1371/journal.pone.0099413 (PMC4059654; doi:10.1371/journal.pone.0099413)
Supplement: File S1 — Supporting material. (PDF) [file pone.0099413.s001.pdf]

Supporting material

Tight coupling of Na^+/K^+-ATPase with glycolysis demonstrated in permeabilized rat cardiomyocytes

Mervi Sepp, Niina Sokolova, Svetlana Jugai, Merle Mandel, Pearu Peterson, Marko Vendelin

SERCA inhibition. In the experiments, respiration was first initiated by 2 mM ATP and, after stable rate was achieved, 1 μM TG, a potent SERCA inhibitor (1, 2, 3), was introduced into the system. We found that, in Ca^{2+} free conditions (the standard setup in our studies), TG does not alter the respiration level . The rate of oxygen consumption after addition of 2 mM ATP was 30.6 ± 13.57 nmol O_2/min mg prot and after subsequent addition of 1 μM TG the respiration rate was 30.8 ± 13.61 nmol O_2/min mg prot (paired T-test $p = 0.51$, $n = 8$), calculated relative change was 0.5 ± 1.9 % ($p = 0.91$) (Fig. 1, the first Ca/TG pair).

TG inhibition was also tested when the free calcium concentration was elevated. Increase in free Ca^{2+} concentration by 100 nM, 200 nM, and 600 nM in separate experiments increased respiration by 13 %, 25 % and 50 % respectively. The respiration rates obtained at 2 mM ATP after addition of different Ca^{2+} concentrations followed by inhibition of 1 μm TG are given in Fig. 1. Addition of 1 μM TG inhibited respiration by $3-7$ % for all non-zero calcium concentrations used. A drop in respiration by TG relative to the rise brought on by calcium was $20-30\%$ for all tested free calcium concentrations. In conclusion, no TG effect on respiration was detected for cardiomyocytes investigated in Ca^{2+} free environment and very modest effect of TG was detected after concentration of Ca^{2+} was increased within the physiological range.

In addition to investigating respiration, we also studied the effect of TG on ATPase activity spectrophotometrically. We performed SERCA inhibition experiments with deactivated mitochondrial respiration and found that TG had a minor effect on the ATPase activity. After incremental inhibition of 2 mM ATP initiated ATPase activity we observed ca 10 % increase in the total ATPase activity (Fig. 2). This increase in activity can be explained by TG induced Ca^{2+} leak from SR (4, 5) leading to the elevation of Ca^{2+} concentration in the cell and activation of myofibrillar ATPase. As a result, the small effect of TG on SERCA is not detectable when total ATPase activity is monitored.

Similarly to respiration experiments, we further studied the TG effect in situations when free Ca^{2+} concentration was increased. We found that raising free Ca^{2+} stepwise to 100 nM, 200 nM and 600 nM increased the ATPase activity by maximally 20 %. Subsequent introduction of TG into the system had a small effect on the ATPase activity (Fig. 2). While 0.1 μM TG addition did not have an effect, increasing the TG concentration created a small but statistically significant drop in relative ATPase activity from 124 ± 22 % at maximal Ca^{2+} to 118 ± 23 % at 0.6 μM TG concentration and 117 ± 24 % at 1.1 μM TG. The ATPase activity rate at 2 mM ATP is 371.4 ± 176 nmol/min mg prot after titration with calcium ATPase activity is 465.4 ± 158 nmol/min mg prot and after subsequent titration with TG the ATPase activity is 436.1 ± 155 nmol/min mg prot.

In summary, we have shown that in Ca^{2+} free conditions SERCA inhibition does not reduce the total ATPase rate or ATPsynthase rate. Thus, the role of this membrane ATPase is minor in our preparation.

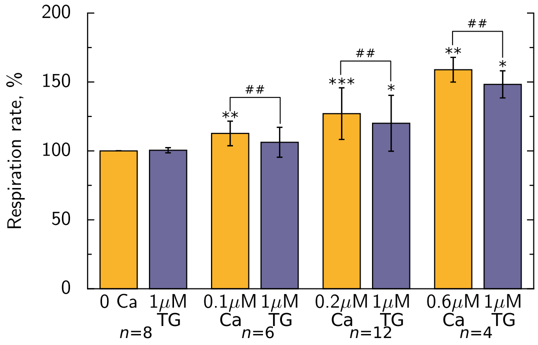

Figure 1: Role of SERCA in respiration. At basal Ca^{2+} concentration, inhibition of SERCA did not affect respiration rate initiated with 2 mM ATP. Increase in free Ca^{2+} produced a rise in respiration rate. SERCA role was evident only in the presence of increased free Ca^{2+} where inhibition of SERCA with TG resulted in less than 10 % drop in respiration. Each pair shows relative change in 2 mM ATP initiated respiration to subsequent addition of Ca^{2+} and TG. When not specified by brackets, statistical significance indicators compare the corresponding result with the rate at Ca^{2+} -free conditions with 2 mM ATP.

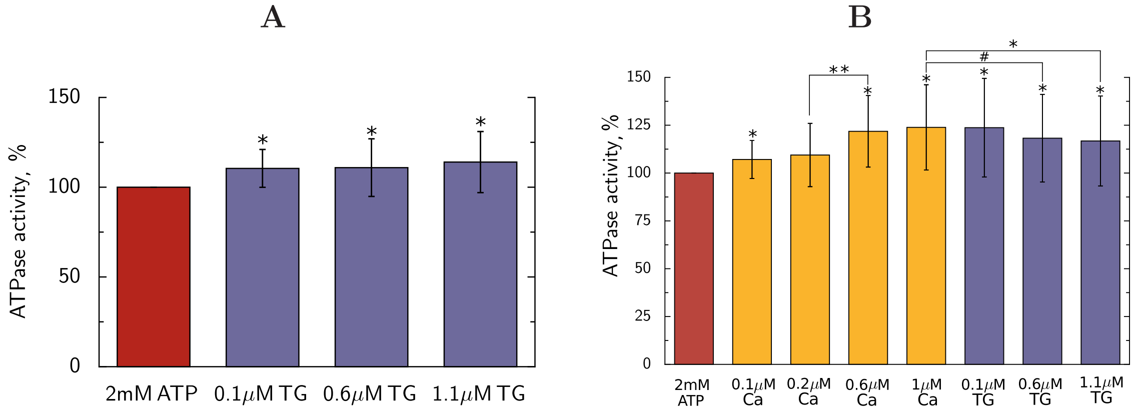

Figure 2: Analysis of SERCA activity in total ATPase activity measured spectrophotometrically using a coupled lactate-dehydrogenase system. A: changes in ATPase activity induced by a stepwise addition of TG at 2 mM ATP. Note that addition of TG does not reduce but instead increases the total ATPase activity (discussed in text); B: To test the SERCA inhibition efficiency, TG was used after free Ca^{2+} levels were increased. Consecutive additions of Ca^{2+} followed by titration with TG relative to the 2 mM ATP induced ATPase activity, n= 12 for both.

References

1. T. B. Rogers, G. Inesi, R. Wade, and W. Lederer. Use of thapsigargin to study ca 2+ homeostasis in cardiac cells. *Biosci. Rep.*, 15(5):341–349, 1995.

2. J. Lytton, M. Westlin, and M. R. Hanley. Thapsigargin inhibits the sarcoplasmic or endoplasmic reticulum ca-atpase family of calcium pumps. *J. Biol. Chem.*, 266(26):17067–17071, 1991.

3. F. Sagara, F. Belda-Fernandez, L. de Meis, and G. Inesi. Characterization of the inhibition of intracellular ca2+ transport atpases by thapsigargin. *J. Biol. Chem.*, 267(18):12606–12613, 1992.

4. L. Hove-Madsen, A. Llach, and L. Tort. Quantification of ca2+ uptake in the sarcoplasmic reticulum of trout ventricular myocytes. *Am. J. Physiol.*, 275(6 Pt 2):R2070–R2080, 1998.

5. L. Song, M. Stern, E. Lakatta, and H. Cheng. Partial depletion of sarcoplasmic reticulum calcium does not prevent calcium sparks in rat ventricular myocytes. *J. Physiol. (Lond.)*, 505(3):665–675, 2004.
